# Choosing a survey sample when data on the population are limited: a method using Global Positioning Systems and aerial and satellite photographs

**DOI:** 10.1186/1742-7622-9-5

**Published:** 2012-09-11

**Authors:** Harry S Shannon, Royce Hutson, Athena Kolbe, Bernadette Stringer, Ted Haines

**Affiliations:** 1 Department of Clinical Epidemiology and Biostatistics, McMaster University, CRL-221, 1280 Main St West, Hamilton, ON, Canada, L8S 4K1; 2 School of Social Work, Wayne State University, Detroit, Michigan, USA; 3 Social Work and Political Science, University of Michigan, Ann Arbor, Michigan, USA; 4 Faculty of Health Sciences, Simon Fraser University, Burnaby, Canada; 5 Present address: Boise State University, Boise, Idaho, USA

**Keywords:** Sampling methods, Surveys, Surveys in difficult situations, Sampling weights, Global Positioning Systems, Aerial photographs, Satellite photographs, Lebanon

## Abstract

**Background:**

Various methods have been proposed for sampling when data on the population are limited. However, these methods are often biased. We propose a new method to draw a population sample using Global Positioning Systems and aerial or satellite photographs.

**Results:**

We randomly sampled Global Positioning System locations in designated areas. A circle was drawn around each location with radius representing 20 m. Buildings in the circle were identified from satellite photographs; one was randomly chosen. Interviewers selected one household from the building, and interviews were conducted with eligible household members.

**Conclusions:**

Participants had known selection probabilities, allowing proper estimation of parameters of interest and their variances. The approach was made possible by recent technological developments and access to satellite photographs.

## Background

Surveys in war zones or other difficult situations have various aims, including estimation of mortality and other harms, population needs and vaccination coverage. Yet such surveys pose considerable challenges for researchers, as there may be little information on which to base the sampling method. Further, in conflict areas risks to the interviewers may limit how information is obtained on the population of interest and how sample data are collected. Particularly when rapid assessment is needed, researchers must balance several desirable properties of surveys: unbiasedness, precision, speed and simplicity.

Various approaches have been used to overcome the challenges and still allow valid calculation of point estimates and their confidence intervals. The crucial requirements for the analysis are that: a) the probability of including a sampling unit can be determined; and b) the design effect (that allows for the sampling process) can be computed. These allow the sampling weights to be applied and proper point and variance estimates to be calculated.

In this paper we introduce a new sampling method which uses Global Positioning System (GPS) technology and aerial/satellite photography. We used this approach when two particular problems applied: information on the target population was limited and it was considered too risky for interviewers to conduct enumerations on site (enumeration entails listing all eligible sampling units – such as individuals or households.) We will discuss the advantages of this new method, in particular, we can account for building density, which is not possible in most previously used methods. We also note some limitations. We begin, though, by describing some methods that have been used, pointing out their strengths and weaknesses.

### Previously used sampling methods

Simple or stratified random sampling is not feasible when there is no enumeration of the target population. Multi-stage sampling may be used if there is limited information on the target population. The method may incorporate clusters, which reduces the cost of interviewing, since the time and expense of travel is reduced. Since more interviews can be conducted for the same cost, the greater sample size typically outweighs the loss of power resulting from the clustering. The method requires dividing the population into distinct clusters, usually based on geography. Available data often allow sampling of clusters using probability proportional to size (PPS); in practice, errors in the cluster size estimates mean the method is really probability proportional to *estimated* size, PPES.

If clusters are small, all units may be included in the sample. If they are larger some method of sampling from within clusters is needed. Ideally, the cluster can be enumerated and a random sample chosen. If this is not feasible, an alternative is to stand in the centre of each cluster and choose a direction randomly, e.g., by spinning a pen. All dwellings from the centre to the edge of the cluster in the chosen direction are counted, one is chosen at random and interviews are conducted. Additional houses are selected along the line away from the centre. If the cluster edge is reached before the sample size is achieved, the interviewers move clockwise to the next house and back towards the centre conducting interviews along the way. Henderson and colleagues, who developed the approach, noted that the method is biased to sampling houses close to the centre. Bias can also result from ‘pocketing’ - uneven spatial distribution of the variables of interest [1].

The Expanded Programme on Immunization (EPI) method identifies the starting house similarly, but then selects other houses by picking the one nearest to the last one included until the cluster sample size is reached [2]. The EPI approach aims to estimate vaccination coverage with a 95% confidence interval (CI) no more than ±10%. It samples 30 clusters of seven eligible subjects each. Two simulations have concluded that overall the method achieves its aims [3,4]. Yet it is not without problems. Identifying houses on a straight line form the cluster centre may be difficult in urban areas. As well, any pocketing may lead to under- or over-estimation of the prevalence in the cluster [5]. Further, any household spatially separate from other households in the cluster could only be included as the starting household, since it would never be the nearest household to any other.

Lemeshow and Robinson noted that interviewer discretion might create bias. It may be easier for the interviewer to identify the starting house without counting all the households to the edge of the cluster or not apply objective distance in choosing the ‘nearest’ household. Finally, the starting household might be chosen for convenience, not at random [5].

Some attempts have been made to improve the EPI method. Choosing the fifth nearest (rather than the nearest) household has been proposed, as has the use of several starting points in different parts of large communities so the sample is spread out [6].

Brogan and her colleagues reminded readers of the concerns about whether the sample within clusters is properly randomin the absence of cluster enumerationRecognising that this may not be feasible, they suggest segmenting the initial clusters into sub-segments so that full enumeration can be done and a proper sample taken [7].

Turner et al. wished to improve on the EPI design, while maintaining a degree of simplicity. They proposed maintaining the PPES of the EPI method followed by sketch-mapping the sample clusters, creating segments of roughly equal size (equal across all selected clusters), randomly choosing one segment per cluster, and interviewing *all* eligible persons within the segments chosen. The method requires knowledge of clusters and their sizes to a fairly fine level – the authors cited a national survey in Bangladesh with PPES sampling of administrative subdivisions, each containing roughly 250–300 households [8].

In two surveys in western Gambia just a few months apart, one adopted the EPI plan, the other used segmenting. The results were similar, but segmenting was recommended as it is less susceptible to poor quality fieldwork and can give estimates of population totals (rather than just proportions and means) to guide planning [9].

‘Spin-the-pen’ selection of the first household was compared with two other approaches. One superimposed a grid on a map of the cluster, randomly chose coordinates on the grid, and identified the closest compound (houses in the setting tended to be in walled compounds). The second used GPS coordinates to identify a randomly chosen point and the nearest compound to the right when facing north at the point. Survey teams found the new methods easier to implement than spin-the-pen. They were most enthusiastic about the GPS method, although the grid approach was fastest. However, both alternative methods led to higher probabilities of choosing households in low density areas of the clusters [10].

Roberts also noted the problem of differing household density. In Katana, Democratic Republic of the Congo (DRC), he used a grid to identify sampling points. For each point the five closest households were selected. Density was allowed for by estimating the ‘radius of the sampling point’ - the distance between the point and the furthest of the five households. The report was short and did not describe just how the radius was incorporated into the analysis [11].

In summary, various methods for sampling in difficult situations have been proposed. Cluster sampling is the basis for most although methods of selecting units within clusters vary – in part because of local circumstances or available data. However, these methods are often biased, unless it is possible to enumerate the cluster population, and they do not take account of housing density in estimating the sampling weight. We build on previous approaches to introduce a new method of sampling within clusters to deal with these problems, given the limited information available on the population and security concerns for interviewers should they try to conduct an enumeration.

## Method of sampling

### Background

The survey was conducted in Southern Lebanon in 2008 to estimate the extent of violence experienced by the population since the war with Israel in 2006, and obtain other information including attitudes to possession of arms by civilians.

### Sampling design

We first selected the towns and villages (henceforth simply ‘towns’). There were three major cities, and we decided we should include them in the survey - we decided to sample 400 households in Tyre, 200 in Marjayoun, and 200 in Bint Jbeil. There were 144 other towns, and by sampling 50 of them and obtaining 16 households per town, we would reach our required sample size of 1,600. We had access to voter rolls to estimate the number of people living in each town or city, although the data were out of date and not reliable. We nevertheless used the data available to sample towns with PPS, which in practice was probability proportional to estimated size (PPES). We were thus selecting a stratified sample, with cluster sampling using PPES in one stratum (the 144 towns), and one-stage spatial random sampling in the other three strata.

### Selection of households within towns

We faced two challenges in identifying our sample. We believed it would be unsafe for interviewers to conduct enumerations, so wanted to limit the time and effort they had to spend in the field. At the same time we did not have data on the populations within towns which we could use to choose the sample.

We obtained pre-2006 geo-coded digital overhead maps (photographs) of the chosen towns. We randomly sampled GPS coordinates within the towns. The corresponding points were located and a circle around each point was drawn on the photograph. The radius represented 20 m on the ground, though this could be varied according to circumstances. Buildings within the circle were numbered, and one was randomly chosen. The method can be seen as similar to that of Grais and colleagues [10], but we accounted for housing density by counting the number of buildings in the circle to obtain the proper sampling weights. If there was no building within the circle, we continued sampling. These activities did not require on-site activity and were conducted before the field work.

Interviewers subsequently used the maps to find the building. If it was not a residential building, the interviewers noted all the buildings within 20 m and then randomly selected one. To do this they were given random number charts for a series of the possible number of buildings/households (up to 16). Going clockwise from the north (demarcated on the map) would then select the building corresponding to the random number.

If there was no ‘valid’ residential building, that point was disregarded. Non-response was rare and occurred for the following reasons:

1. The occupants were living abroad - outside Lebanon (N = 9; 0.6%).

2. Households were not occupied and no information could be obtained about the residents (N = 32; 2.0%).

3. Households were not occupied and there were no nearby houses where we could ask about the residents of the sampled household (N = 19; 1.2%).

When the interviewers identified the residential building, they checked if there was one residence or more than one. In the latter case, they used pre-prepared random number tables to select one residence. They requested an interview with one resident adult (18 years old or older), the one with the most recent birthday. This person was asked to answer some questions on behalf of the household and other items based on his/her own attitudes and experiences (e.g., questions about post-traumatic stress disorder). Each interview was attempted up to four times before noting a non-respondent household. We continued to sample households via GPS locations to achieve the required sample size in each town, and kept a record of how often replacement households had to be recruited.

All buildings were clearly marked on the maps. Most villages in South Lebanon are small, so locating a house with clear landmarks nearby was not difficult. In the urban settings, Sour (Tyre) and Marjaoun, location was more challenging. Interviewers counted from the corners of streets to verify that the correct building was identified.

### Sample size

Our primary objective was to estimate the proportion of people suffering some violation. A priori we believed that 20% was a reasonable estimate. A completely random sample would have needed 1600 respondents to obtain a 95% confidence interval (CI) of ±2%. We assumed that the effect of clustering would no more than double the width of the CI, so our CI would be ±4%, satisfactorily narrow.

### Analysis and weights

The analysis had to allow for the sampling method, in particular, the sampling of clusters (towns) and the different probabilities of selection of households and individuals.

#### Probability of sampling each town

We used PP(E)S to identify towns (other than the automatically included cities of Tyre, Marjayoun, and Bint Jbeil). The probability of sampling a particular town was labeled *p*_*1*_*.*

#### Probability of sampling households and individuals

Within towns, the GPS locations defined a series of circular areas. After the interviews had been completed, we determined how many locations had had to be sampled (including those containing no residential building) to achieve the sample for that town. The total area of all the circles at these locations divided by the area of the town was labeled *p*_*2*_.

For each circle containing residential buildings, the inverse of the number of buildings was labeled *p*_*3*_. The number of separate units (households) in the selected building was determined and its inverse was labeled *p*_*4*_. Finally, the inverse of the number of adults in the household was labeled *p*_*5*_.

The product p_h_ = p_1_p_2_p_3_p_4_ was the probability that the household was chosen. The product p_r_ = p_1_p_2_p_3_p_4_p_5_ was the probability that the respondent individual was chosen. The analysis took account of the sampling method. The towns were treated as clusters and sampling weights were the inverses of the probabilities of selection.

### Ethics

The protocol was approved by the Institutional Review Board at Wayne State University and Lebanese American University, Beirut, Lebanon.

## Discussion

We have proposed a novel way to take a sample when there is limited information on the population under study. It uses technology and data (Global Positioning Systems and satellite and aerial photographs) that are now widely available, and overcomes problems with other approaches.

The method has several strengths: it reduces the work for interviewers, minimizes their discretion in choosing buildings and is safer for them. It allows random selection with known probabilities, and minimizes ‘pocketing’ within clusters by spreading out the sample within the cluster. Unlike many previous techniques, it incorporates population (household) density, which permits calculation of correct sampling probabilities. Enumeration of buildings is needed for only very small areas, a task that can be done before going into the field; and interviewers only need to enumerate households for multi-residential buildings.

Given our experience, we raise several other issues. The latest satellite or aerial photographs for the GPS locations available to researchers can be out of date; interviewers should confirm the correct number of buildings when they visit the location. In our survey, we deliberately used older photos (before July 2006), since we wanted to learn about people who had left the area or had their homes destroyed. Most surveys will require recent photos of sufficient resolution to discern between buildings in dense areas. We used two separate mapping tools, both geocoded, that were recent aerial photos of the areas covered. We used both Google Earth and maps obtained from a local aerial mapping firm (in ArcGIS formatting) that had conducted a survey of the region less than a year before the conflict. Google Earth photos had been taken on May 31, 2006, less than 1.5 months before the onset of hostilities. In the cases in which resolution was poor, as in some rural areas (an issue only for Google Earth), the maps were cross-referenced for accuracy in detail. The resolution of the privately purchased maps was often significantly better than Google Earth’s photos, in which case we used the former.

There is also the question of defining when a building is ‘in’ the circle surrounding the GPS point – what part / proportion should be inside the circle. We recommend basing this decision on the ‘centre’ of the building; irregular shapes might cause error, though this is likely negligible.

We used circles with radius 20 m. In practice, the length of the radius may depend on the density of buildings in the areas under study. The circles surrounding two (or more) GPS points might overlap. Strictly, adjustments need to be made in computing both the probability of selecting buildings in the overlap and the fraction of the town area covered by the circles surrounding the points. Some preliminary simulations suggest any biases from failing to do this are minimal. This does depend partly on the area of the town and the number and radius of the circles, since they determine the likelihood of overlap. An alternative that can prevent this problem is to adapt the grid approach others have used [e.g., Grais et al., 2007]. On a map of the area under study, one could superimpose a grid of non-overlapping squares. Then a defined number of squares could be randomly sampled, and as with circles, buildings can be enumerated and one randomly chosen. (The question of whether a building is truly inside a square still applies.)

Another possible amendment to our method deals with the question of what to do if the building chosen is non-residential. Rather than ask interviewers to identify residential buildings within 20 m and randomly choose one, before the interviews begin one could designate 2^nd^ or 3^rd^ choice buildings within the circle. This would reduce the work of interviewers and limit their discretionary decisions.

The safety and security of interviewers needs to be maintained, even at the expense of efficiency of the design or complete adherence to protocol. This was done in a survey in Iraq [12]. We too were concerned that outside interviewers might be at some risk; for example, that they might be seen as spies and a priori we excluded two Palestinian refugee camps. We also deemed it imprudent for interviewers to map out the boundaries of the selected towns on site. Indeed, because of intervention by Hizbollah security personnel, we were not allowed to conduct the survey in Bint Jbeil, where we had anticipated surveying 200 households, and Khiam. Since the region of these towns was one of the hardest hit during the conflict, we likely underestimated numbers of casualties and rights violations. As well, interviewers could find locations with GPS units rather than satellite photographs. We did not do this, as we were worried about the safety of interviewers if they were known to be using GPS technology. The more recent availability of ‘smart’ phones with GPS capability may circumvent this concern, as observers might simply assume the interviewers were using their phones.

As well, talking with local leadership about the study, in particular the nature of the maps and the random choice of locations, before conducting interviews decreased the amount of suspicion and increased acceptance of the survey teams by local residents. Even though we did this through our local interviewing firm to great effect, we were not allowed into two of the strongholds of Hizbollah, whose local leaderships’ biggest concern was the nature of the questions on ownership of and attitudes towards small arms.

It may be feasible to adjust estimates using alternative sources. For example, the Iraq Body Count has collected data on the numbers killed, as reported in newspapers or other sources [13]. By using data on the relative proportions of people reported killed in different cities, the Iraq Family Health Survey Study Group estimated the undercount in their survey [14]. We did not have relevant information, so had to accept the limitations in our data from failure to cover the whole area.

We recognize that many agencies planning surveys have limited expertise and resources. Google Earth is free and readily available with internet access. Agencies, we believe, will find the tool very attractive for this reason. In addition, random selection of GPS coordinates can easily be conducted in almost any statistical package or spreadsheet application, including Excel. In addition, importing those points into Google Earth tools can be conducted easily with open access free software (e.g., GPS Visualizer: http://www.gpsvisualizer.com/map_input?form=googleearth).

Though the technical expertise necessary to carry out these processes may seem daunting for some agencies, we believe with a limited amount of training most program officials will be able to easily and quickly use this process in emergency and/or difficult settings. Using Google Earth is intuitive and can be learned quickly. Additionally, training in randomly selecting GPS coordinates and mapping them to the software should be relatively brief. Once that is done, Google Earth tools can be used to delineate the 20 m radius for each point, demarcating the buildings, and randomly selecting one. The maps are then printed directly from the program and given to the interviewers. In an Appendix, we show the calculations needed to compute sample weights, and the syntax for doing this in SPSS, a widely used statistical package.

## Conclusions

We have described a novel method of sampling for a survey when only limited information is available on the population being studied and when it is not feasible to enumerate even subsets of the population. Recent developments in technology and access to satellite photographs have allowed us to develop this extension of other approaches reported in the literature, which overcomes difficulties of those approaches. The method proved feasible in a difficult situation, although some limitations, both practical and theoretical, have been noted. We hope others will use and improve on our approach.

## Appendix

### A.1. How to analyze data using the method described in the paper

We show an example in which towns are selected by PP(E)S, GPS locations are sampled in the towns selected, one building is chosen from each circle surrounding the GPS point, one household is sampled from each building, and one adult in each household is asked about him/herself and about characteristics of the household.

Information/variables needed – these are entered into the appropriate field for each respondent as shown in Table 1:

**Table 1:** Descriptions and labels of variables needed for the computations

**Variable description**	**Variable label**
(Estimated) Population of the town in which the person resides	TownSize
Total population size (constant across the study)	TotPop
Area of the town in square metres (= 10^6^ * area in square km)	Area
Radius of circle from which the household was selected	Radius
Number of circles needed for the town to achieve the specified cluster sample size (including circles containing no building or no residence)	Circles
Town code	Town
Number of buildings in the circle	Buildings
Number of households in the building	Households
Number of (eligible) adults in the household	Adults

NOTES:

These variables are entered into the information on each individual/household in the study. The first five items are obtained before the interviewers conduct the survey. The number of buildings is confirmed by the interviewers, who also determine the last two variables.

The populations of each town, including those not selected for the sample, are added to produce the total population size.

The radius of the circles is likely to be fixed. In practice, it could be varied, and the appropriate value inserted for each individual/household interviewed.

We can estimate:

p1 = TownSize / TotPop = the proportion of the overall population in the town.

p2 = Circles * Radius**2 * 3.14159 / Area = Proportion of the area of the town covered by the circles.

p3 = 1 / Buildings = Probability of selecting the building chosen from buildings in the circle.

p4 = 1 / Households = Probability of selecting the Household chosen from households in the building.

p5 = 1 / Adults = Probability of choosing the adult from adults in the household.

Then the overall probabilities of selecting the households and individuals are:

pH = p1 * p2 * p3 * p4 = Probability of choosing the household.

pA = pH * p5 = Probability of choosing the adult.

These lead to the sampling weights

wH = 1 / pH = Sampling weight for the household.

wA = 1 / pA = Sampling weight for the individual interviewed.

**SPSS syntax** required to compute these variables (using the variable names in Table 1):

DATASET ACTIVATE DataSet.

COMPUTE p1 = TownSize / TotPop.

COMPUTE p2 = Circles*Radius ** 2 * 3.14159/Area.

COMPUTE p3 = 1/Buildings.

COMPUTE p4 = 1/Households.

COMPUTE p5 = 1/Adults.

COMPUTE pH = p1*p2*p3*p4.

COMPUTE pA = pH*p5.

COMPUTE wH = 1/pH.

COMPUTE wA = 1/wA.

EXECUTE.

Clustering is taken into account by using considering Town as the variable that designates the clusters and wH as the sample weight for items asking about the household, and wA as the sample weight for items asking about the individual interviewed.

## Competing interests

The authors declare that they have no competing interests.

## Authors’ contributions

All authors originated the study, developed the methods and contributed to critical revisions of the article. HS drafted the article. RH led the field work and AK trained the interviewers. All authors read and approved the final manuscript.

## Authors’ information

Harry Shannon and Ted Haines are with the Department of Clinical Epidemiology and Biostatistics, McMaster University, Hamilton, Ontario, Canada. At the time of this project, Royce Hutson was with the School of Social Work, Wayne State University, Detroit, MI, and is now at Boise State University, Boise, ID. Athena Kolbe is a student in the University of Michigan joint Social Work and Political Science doctoral program. Bernadette Stringer is with the Faculty of Health Sciences at Simon Fraser University, British Columbia, Canada.
